# Supplementary material for: Multi‐dataset identification of innovative feature genes and molecular mechanisms in keratoconus
Source: J Cell Mol Med. 2024 Sep 19;28(18):e70079. doi: 10.1111/jcmm.70079 (PMC11412914; doi:10.1111/jcmm.70079)
Supplement: Supplementary file 2 — Material S2. [file JCMM-28-e70079-s002.zip › 7.GSEA analysis/4.MYRF/KEGG_PORPHYRIN_AND_CHLOROPHYLL_METABOLISM.html]

Details for gene set KEGG\_PORPHYRIN\_AND\_CHLOROPHYLL\_METABOLISM[GSEA]

|  || Dataset | MYRF.group.cls#h\_versus\_l |
| Phenotype | group.cls#h\_versus\_l |
| Upregulated in class | h |
| GeneSet | KEGG\_PORPHYRIN\_AND\_CHLOROPHYLL\_METABOLISM |
| Enrichment Score (ES) | 0.43660814 |
| Normalized Enrichment Score (NES) | 1.3998402 |
| Nominal p-value | 0.08280255 |
| FDR q-value | 1.0 |
| FWER p-Value | 0.884 |
Table: GSEA Results Summary

  

Fig 1: Enrichment plot: KEGG\_PORPHYRIN\_AND\_CHLOROPHYLL\_METABOLISM      
 Profile of the Running ES Score & Positions of GeneSet Members on the Rank Ordered List

  

| SYMBOL | TITLE | RANK IN GENE LIST | RANK METRIC SCORE | RUNNING ES | CORE ENRICHMENT || 1 | UGT1A8 | na | 369 | 0.620 | 0.1819 | Yes |
| 2 | UGT1A6 | na | 2556 | 0.320 | 0.2183 | Yes |
| 3 | UGT2B4 | na | 3822 | 0.260 | 0.2626 | Yes |
| 4 | MMAB | na | 5270 | 0.214 | 0.2874 | Yes |
| 5 | ALAS2 | na | 5495 | 0.208 | 0.3454 | Yes |
| 6 | UGT2B11 | na | 6521 | 0.178 | 0.3710 | Yes |
| 7 | ALAD | na | 6816 | 0.169 | 0.4151 | Yes |
| 8 | UGT2B28 | na | 7657 | 0.147 | 0.4366 | Yes |
| 9 | CPOX | na | 10254 | 0.091 | 0.3901 | No |
| 10 | UGT2B10 | na | 12166 | 0.061 | 0.3540 | No |
| 11 | FECH | na | 12255 | 0.060 | 0.3700 | No |
| 12 | HMOX2 | na | 14953 | 0.028 | 0.3011 | No |
| 13 | UGT2A3 | na | 16075 | 0.017 | 0.2741 | No |
| 14 | UGT2B7 | na | 16473 | 0.013 | 0.2667 | No |
| 15 | UROS | na | 16514 | 0.013 | 0.2696 | No |
| 16 | EARS2 | na | 16578 | 0.012 | 0.2716 | No |
| 17 | PPOX | na | 16635 | 0.012 | 0.2736 | No |
| 18 | FTMT | na | 16883 | 0.010 | 0.2695 | No |
| 19 | BLVRB | na | 17658 | 0.003 | 0.2480 | No |
| 20 | UROD | na | 17689 | 0.002 | 0.2479 | No |
| 21 | HMBS | na | 17789 | 0.001 | 0.2455 | No |
| 22 | BLVRA | na | 17985 | -0.000 | 0.2399 | No |
| 23 | GUSB | na | 18367 | -0.004 | 0.2302 | No |
| 24 | FTH1 | na | 18527 | -0.005 | 0.2273 | No |
| 25 | COX15 | na | 21160 | -0.035 | 0.1624 | No |
| 26 | CP | na | 22642 | -0.054 | 0.1366 | No |
| 27 | HCCS | na | 22876 | -0.058 | 0.1478 | No |
| 28 | HMOX1 | na | 23761 | -0.072 | 0.1446 | No |
| 29 | ALAS1 | na | 24023 | -0.076 | 0.1608 | No |
| 30 | UGT2B15 | na | 26025 | -0.113 | 0.1383 | No |
| 31 | COX10 | na | 27866 | -0.157 | 0.1340 | No |
| 32 | UGT2A1 | na | 29490 | -0.205 | 0.1510 | No |
Table: GSEA details [plain text format]

  

Fig 2: KEGG\_PORPHYRIN\_AND\_CHLOROPHYLL\_METABOLISM      
 Blue-Pink O' Gram in the Space of the Analyzed GeneSet

  

Fig 3: KEGG\_PORPHYRIN\_AND\_CHLOROPHYLL\_METABOLISM: Random ES distribution      
 Gene set null distribution of ES for **KEGG\_PORPHYRIN\_AND\_CHLOROPHYLL\_METABOLISM**
